# Y44A Mutation in the Acidic Domain of HIV-2 Tat Impairs Viral Reverse Transcription and LTR-Transactivation

**DOI:** 10.3390/ijms21165907

**Published:** 2020-08-17

**Authors:** Zsófia Szojka, János András Mótyán, Márió Miczi, Mohamed Mahdi, József Tőzsér

**Affiliations:** 1 Laboratory of Retroviral Biochemistry, Department of Biochemistry and Molecular Biology, Faculty of Medicine, University of Debrecen, 4032 Debrecen, Hungary; szojka.zsofia@med.unideb.hu (Z.S.); motyan.janos@med.unideb.hu (J.A.M.); miczimario@med.unideb.hu (M.M.); 2 Doctoral School of Molecular Cell and Immune Biology, University of Debrecen, 4032 Debrecen, Hungary

**Keywords:** human immunodeficiency virus, HIV-2, Tat, stability analysis, reverse transcriptase activity, transactivator protein, mutation design

## Abstract

HIV transactivator protein (Tat) plays a pivotal role in viral replication through modulation of cellular transcription factors and transactivation of viral genomic transcription. The effect of HIV-1 Tat on reverse transcription has long been described in the literature, however, that of HIV-2 is understudied. Sequence homology between Tat proteins of HIV-1 and 2 is estimated to be less than 30%, and the main difference lies within their N-terminal region. Here, we describe Y44A-inactivating mutation of HIV-2 Tat, studying its effect on capsid production, reverse transcription, and the efficiency of proviral transcription. Investigation of the mutation was performed using sequence- and structure-based in silico analysis and in vitro experiments. Our results indicate that the Y44A mutant HIV-2 Tat inhibited the activity and expression of RT (reverse transcriptase), in addition to diminishing Tat-dependent LTR (long terminal repeat) transactivation. These findings highlight the functional importance of the acidic domain of HIV-2 Tat in the regulation of reverse transcription and transactivation of the integrated provirions.

## 1. Introduction

The human immunodeficiency viruses type 1 and 2 (HIV-1, and HIV-2, respectively) are the causative agents of the acquired immunodeficiency syndrome (AIDS). These viruses share a similar genomic organization, consisting of structural genes, such as the *gag*, *pol*, and *env* genes, as well as regulatory and accessory genes. While the accessory genes (*nef*, *vpr*, *vpx*, and *vif*) have been attributed to increased viral infectivity, regulatory proteins, such as the transcriptional transactivator (Tat) and transactivating Rev proteins, are required for efficient viral replication [1,2]. Replication dynamics of HIV-1 and HIV-2 are thought to be similar; however, the clinical course of HIV-2 infection differs from that of HIV-1. Plasma viral load and DNA load in patients infected with HIV-2 are considerably lower, indicating a significantly decreased rate of replication [3], which can be attributed to a significantly lower level of *tat* mRNA transcripts [4]. Furthermore, the effective number of codons was found to be higher in the *tat* gene of HIV-2 compared to HIV-1, reflecting a decreased expression of HIV-2 Tat, which may be partly responsible for the lower viral load observed in HIV-2 infected subjects [5]. Moreover, a major difference between the two viruses lies within the structure of their long terminal repeat (LTR) regions. LTRs in HIV-2 are significantly larger, and contain complex structures that are not present in those of HIV-1 [6].

Tat is an essential, multifunctional transactivator in the viral life-cycle, activating proviral gene expression by inducing remodeling of the chromatin, and regulating cellular transcription factors [7,8,9]. Regulation of viral genome expression is mediated via binding to a transactivation response (TAR) RNA element on the LTR promoter, which induces elongation [10,11]. Tat also stimulates reverse transcription through interaction with cellular positive transcription elongation factor b (P-TEFb); composed of cyclin T1, cyclin-dependent kinase 9 (CDK9) and histone deacetylases [7,8], forming a huge complex with several human transcription factors/coactivators such as AFF4, ENL, AF9, and ELL2 [12]. The Tat:P-TEFb complex controls/regulates RNA polymerase II (RNAPII) [13], transcription elongation factor Spt5, and negative elongation factor E (NELF-E) by hyperphosphorylation [14].

Previous studies on HIV-1 Tat protein demonstrated that Tat stimulates reverse transcription though direct interaction with p51 and p66 subunits of RT (reverse transcriptase) [15], and also by enhancing the binding of RT to the RNA template [16], as well. While wild-type Tat stimulates the activity of RT, Nullbasic Tat mutations; where the entire basic domain is replaced by Gly and Ala residues, and Y47D substitution [17], were found to hinder Tat-mediated transactivation, extinguish reverse transcription, and inhibit the activity of Rev [18,19,20].

There is less than 30% identity in the amino acid sequence between Tat of HIV-1 and that of HIV-2 [21], however, certain regions show high similarity. The length of HIV-1 Tat (86–104 residues) may vary among different HIV-1 strains [22,23], molecular weight of which is estimated to be between 9–14 kDa [9]. In most HIV-1 isolates, Tat consists of 101 residues [24], whereas that of laboratory-adapted HIV-1 isolates such as HIV-1 HXB2, LAI, and BRU strains used in in vitro experiments, contain 86 amino acids [13]. On the other hand, Tat of HIV-2 is substantially longer, consisting of 130 residues, and its molecular weight ranges from 14 to 16 kDa [25,26].

**Figure 1:**
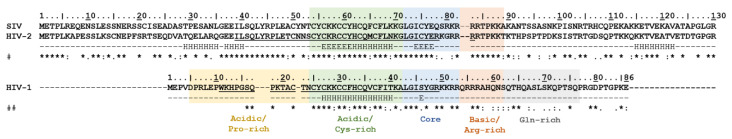
Sequences of SIV (simian immunodeficiency virus), HIV-1, and HIV-2 Tat (transactivator) proteins. Alignment of SIV, HIV-2 and HIV-1 Tat was performed using the following sequences: HIV-2 Tat, P04605; SIV Tat, M33262. HIV-1 Tat domains are indicated based on data published previously [28], while HIV-2 Tat domains are shown based on similarities to HIV-1. Regions predicted to be globular are underlined within the sequences, and predicted secondary structures are also shown (H: α-helix, E: β-strand). ^#^ and ^##^ indicate that Tat sequences of HIV-2 + SIV, and HIV-2 + HIV-1 were aligned.

Tat proteins of both types of HIV are encoded by two exons. Exon I plays a main role in transcription, while exon II exerts an NF-*κ*B-dependent control of HIV-1 transcription and enhances HIV replication in T cell lines. It is also essential to the repression of the expression of major histocompatibility (MHC) class I [7,27]. In HIV-1, Tat protein contains the following domains: an N-terminal acidic Pro-rich, an acidic Cys-rich, a hydrophobic core, a basic Arg-rich, and a C-terminal Gln-rich domain [28] (Figure 1). While the first five domains are encoded by the first exon, the sixth domain is encoded by the second exon [29].

HIV-2 Tat has a longer N-terminal region, which contains fewer Pro residues than the Pro-rich acidic domain of HIV-1 Tat, and shares only a 10% homology with the same domain of its counterpart [30]. The Cys-rich, core, and the Arg-rich domains show relatively high sequence identity between Tat proteins of HIV-1 and HIV-2, while a Gln-rich domain can only be found in Tat of HIV-1 (Figure 1).

While single-residue changes in the N-terminal proline-rich region of HIV-1 Tat, which contains conserved residues including W11 [20] and acidic amino acids, exert no effect on the protein’s function and can be well tolerated [24,25], mutations and deletions in the Cys-rich domain and hydrophobic core were found to be detrimental to transactivation [31]. It was previously reported that the transcription activation function of HIV-1 Tat can be abolished upon Y26A [32] and SIV (simian immunodeficiency virus) Tat by Y55A [33] amino acid substitutions. Y55 residue of SIV and HIV-2 Tat proteins corresponds to the highly conserved Tyr residue within the Cys-rich domain at the 26th position of HIV-1 Tat (Figure 1).

Notably, to the best of our knowledge, mutations in the acidic domain of HIV-2 Tat have not yet been functionally characterized, and their effect on reverse transcription has not been determined, keeping in mind that the N-terminal region of HIV-2 Tat shows only a 10% homology with that of HIV-1 [30]. Although previous studies have focused on the Cys-rich domain of Tat in HIV-1 and SIV, the effect of Y44A substitution in the first domain of HIV-2 Tat remains to be elucidated.

Recently, we conducted dual infection experiments to study the interactions between HIV-1 and HIV-2 [34]. We found that pre-transduction of cells with HIV-2 protects against HIV-1 superinfection. While investigating the putative role of HIV-2 accessory and regulatory proteins in this dampening of HIV-1′s infectivity as a result of pre-transduction with HIV-2, we carried out a series of mutagenesis. Among them, we found that Y44A mutation completely inactivated HIV-2 Tat. On a similar note, Y55A mutation in the Cys-rich domain of SIV Tat protein was also reported to inactivate the protein [33].

Here, we present a sequence- and structure-based mutation analysis supported by in vitro characterization of the Y44A mutant HIV-2 Tat protein. The effects of protein inactivation on viral capsid production, RT activity, and transduction efficiency were compared to those of the wild-type and Y55A mutant proteins. The results of our study indicate that Y44A mutation in the N-terminal Pro-rich domain severely limits the transactivation function of HIV-2 Tat protein.

## 2. Results

### 2.1. Mapping Destabilizing Mutations

Effects of mutations on HIV-2 Tat protein were analyzed by in silico methods. Predictions were performed based on protein sequences and homology model structures, in order to identify a residue to be targeted by site-directed mutagenesis.

Disorder prediction suggested a globular nature of the central region in HIV-2 Tat (39–78 residues), while both N- and C-terminal regions were predicted to be unstructured. Moreover, the presence of secondary structural elements was also predicted mainly for the central region (29–77 residues). A structural study showed a flexible nature of the Cys-rich domain in HIV-1 Tat [35], while our predictions implied that the region prior to the Cys-rich domain in HIV-2 Tat may be structured (Figure 1).

**Figure 2:**
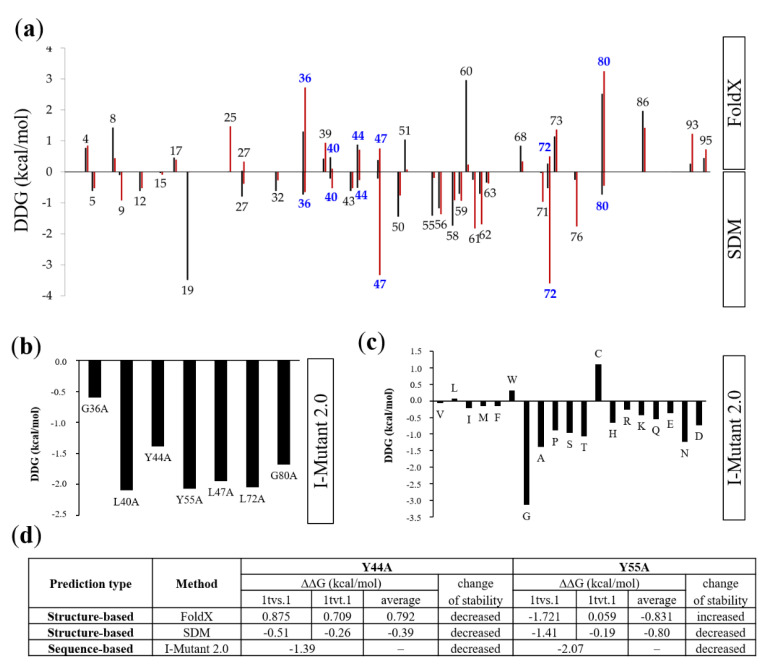
Stability analysis of mutations. (**a**) Graph shows results of alanine-scanning performed by FoldX and SDM (Site Directed Mutator). The free energy differences (DDG, kcal/mol) between wild-type and mutant proteins were plotted only for those residues for which the mutation was considered to be destabilizing in case of both model structures (by black and red bars for 1tvs.1 and 1tvt.1 models, respectively). Alanine substitutions causing DDG > 0 kcal/mol change in stability are considered to be destabilizing in FoldX, while DDG < 0 kcal/mol change implies a destabilizing nature of the mutations in SDM. Blue color highlights those residues of the globular region of which the mutation was predicted to be destabilizing by both methods. (**b**) A sequence-based method (I-Mutant 2.0) was used to predict the effect of alanine substitutions of residues which have been selected based on results of structure-based stability analysis (see figure part A). Negative values (kcal/mol) indicate a destabilizing nature of the mutations. (**c**) Alanine-scanning of Y44 residue by I-Mutant 2.0. Negative values (kcal/mol) indicate a destabilizing nature of the mutations. (**d**) Comparison of the predicted effect of Y44A and Y55A mutations. Values obtained for both homology model structures are shown (SWISS-MODEL Template Library identifiers: 1tvs.1 and 1tvt.1). Stability change was considered to be increased or decreased, based on average values. “‒” indicates that only a single value was obtained, thus, average was not calculated.

To map the potentially destabilizing mutations, sequence- and structure-based methods were applied (Figure 2). While no crystal or nuclear magnetic resonance (NMR) structures are available for the HIV-2 Tat protein, homology models freely accessible in the SWISS-MODEL repository were used. Model structures were available only for truncated proteins, thus, we have chosen those models which contained the entire region that was predicted to be globular.

Structure-based alanine-scanning was performed using FoldX algorithm and Site Directed Mutator (SDM) server, as well. Only those point-mutations were considered to be potentially destabilizing if both methods predicted destabilizing nature of the given amino acid substitution. G36, L40, Y44, L47, L72, and G80 residues fit to this criteria (Figure 2a), the result of structure-based prediction was in good agreement with that of sequence-based analysis performed by I-Mutant server (Figure 2b). All these residues are located within, or in the close proximity of the predicted globular region of HIV-2 Tat, these predicted mutations were assumed to potentially alter the protein structure. Out of the above mentioned six residues, we assumed that Y44A mutation may have the most remarkable effect destabilizing the protein. Mutation of the large and polar Tyr sidechain (Y44A) to a small hydrophobic Ala was predicted to result in a loss of potentional hydrogen bond interactions. In contast, glycine (G36 and G80) and hydrophobic leucine residues (L40, L47, and L72) cannot form such side-shain-mediated polar interactions. Additionally, more remarkable decrease of protein stability was predicted for Y44A and Y44G mutations as compared to other mutations (Figure 2c).

### 2.2. In Silico Analysis

The inactivating effect of Y55A mutation on Tat protein was previously reported for SIV [33], therefore, the effects of the Y44A point mutation were compared to those of the previously characterized Y55A.

Both Y44 and Y55 residues are located in the N-terminal acidic region of HIV-2 Tat, and Y55 is equivalent to the highly conserved Y26 residue of HIV-1 Tat (Figure 1).

**Figure 3:**
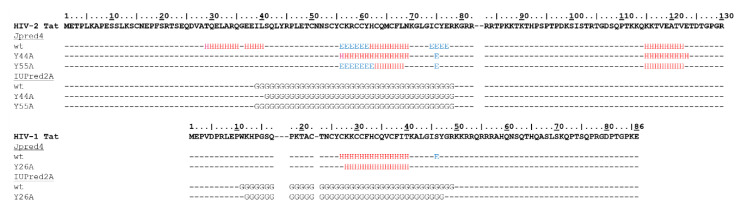
Effects of Y44A and Y55A mutations on secondary structure and globular regions. Amino acid sequences of the wild-type HIV-1 and HIV-2 Tat proteins are shown. Predicted secondary structures are shown for the wild-type and mutant proteins, H (red) and E (blue) denote α-helices and β-strands, respectively (Jpred4). Residues predicted to constitute globular regions (IUPred2A) are shown by G.

Secondary structure prediction implied more remarkable effects of Y44A mutation, compared to that of Y55A. While we predicted putative disruption of the α-helix at the N-terminal part of the globular region for both mutations, predictions showed a higher probability for helical arrangement of the Cys-rich domain as a result of Y44A mutation (Figure 3).

The effects of point mutations on protein stability were also compared, and were found to be similar in the case of Y44A and Y55A mutations (Figure 2d). Values determined by three different methods showed decreased protein stability for the Y44A mutant, based on analysis of both model structures. While FoldX prediction implied increased stability for Y55A mutation, the destabilizing changes in Y55A mutant were predicted to be more remarkable as compared to Y44A.

Y26A and Y55A mutations were previously found to result in the inactivation of HIV-1 and SIV Tat proteins, respectively [32,33]. Both residues are highly conserved based on available data in the HIV Sequence Compendium (2018; https://www.hiv.lanl.gov). Similarly to Y26 of HIV-1 Tat, HIV-2 Tat also contains conserved Tyr residue in the corresponding position of the Cys-rich region (Y55). The Y44 residue of HIV-2 Tat shows high conservation in group B (Figure S1c); however, His and Phe variations were observed in group A (epidemic) and non-epidemic strains (Figure S1b,d).

The predicted destabilizing nature of alanine substitution, and the conserved nature of the Tyr residue in the 44th position, implied that Y44A mutation may potentially be able to induce adverse changes to protein structure, and result in the inactivation of HIV-2 Tat. To explore the effects of Y44A mutation on the protein function, an HIV-2 vector carrying the modified *tat* gene was prepared and used for in vitro experiments. Furthermore, a Y55A mutant was also studied for comparison.

### 2.3. In Vitro Characterization of HIV-2 Tat Mutations

For in vitro experiments, we utilized a ROD strain-based lentiviral vector (HIV-2 CGP) coding for wild-type HIV-2 Tat. Site directed mutagenesis was then carried out on the HIV-2-CGP to inactivate Tat protein, and its success was verified by sequencing.

Initially, we tested the transfection efficiency of the vectors with the wild-type and mutant *tat*, and the transduction efficiency of the pseudovirions in HEK293T cells. Fluorescence-activated cell sorting (FACS) and fluorescent microscopy were used to analyze the transfection efficiency, which ranged from 70–75% for both vectors carrying mutant *tat* (Y44A and Y55A), similar to that observed for the wild-type vector.

Pseudovirion production yielded viral titers of 16–18 ng/mL for wild-type and mutant virions, as determined by a colorimetric SIV p27 assay, and no significant difference was observed in terms of the amount of capsid between the HIV-2 wild-type and Y44A/Y55A Tat mutant pseudovirions. It is worth mentioning that a similar viral titer was obtained for mock pseudovirions containing the HIV-2-CRU5SIN-WPRE plasmid which was used in HIV indicator cell (GHOST) assay.

Transduction efficiency of the pseudovirions in HEK293T cells using 6 ng of viruses (normalized for capsid) was confirmed by flow cytometry, and no significant difference in green fluorescent protein (GFP) expression was observed between the wild-type and mutant Tat pseudovirions. Percentage of fluorescent cells indicating successful transduction did not change significantly upon Y44A and Y55A mutations, as compared to the wild-type HIV-2 (*p* values 0.95 and 0.44, respectively). This was expected, since GFP expression in the HIV-2-CRU5SIN-CGW is driven under a CMV (cytomegalovirus) promoter. A virus carrying an inactivating mutation at the active site of the protease (D25N) was used as a negative control, which resulted in a complete abolishment of viral infectivity (Figure S2).

#### 2.3.1. Experiments in HIV Indicator Cells

**Figure 4:**
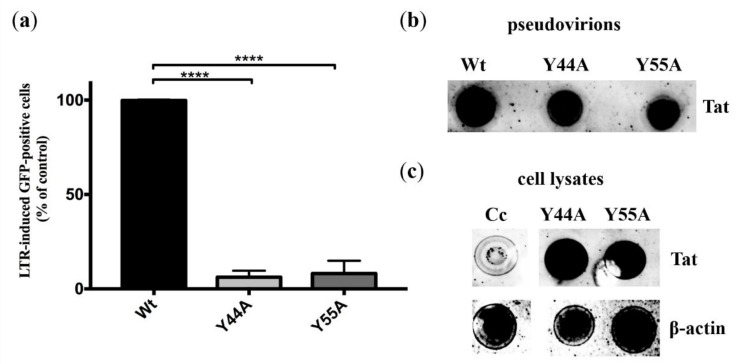
Experiments on HIV-indicator cells. (**a**) GHOST(3) cells were transduced using equal amounts of p27 (5 ng as determined by capsid ELISA) (n = 9). HIV LTR (long terminal repeat)-induced GFP (green fluorescent protein) expression in the presence of HIV-2 wild-type (Wt) and Tat mutations was measured by flow cytometry. GFP-positivity measured for GHOST(3) cells that were transduced with wild-type HIV-2 pseudovirions was considered to be 100%, significantly decreased positivity was determined for Tat Y44A and Y55A mutants (**** *p* value < 0.0001) (n = 9). Error bars represent SD. Wt: wild-type. (**b**) Dot-blotting of lysed pseudovirions was used to demonstrate the presence of Tat protein. (**c**) GHOST(3) cells were transduced with 5 ng Tat-mutant pseudovirions, cells were then lysed, and anti-Tat serum was used to detect Tat from transduced cell’s lysate. β-actin was used as control for cell lysate of transduced GHOST(3) cells. Cc, uninfected GHOST(3) cells.

HIV indicator GHOST(3) cells contain a *tat*-dependent HIV-2 LTR-GFP construct, therefore, a GFP fluorescence is obtained in response to transduction with a functional Tat. Following transduction with 5 ng (normalized for capsid) of wild-type or Tat-mutant pseudovirions, GFP positivity was analyzed by flow cytometry 3 days after transduction. GFP fluorescence signal indicating transactivation was significantly decreased by more than 93% and 91% in the presence of HIV-2 Tat Y44A and Y55A mutations, respectively (*p* value < 0.0001), compared to that of the wild-type (Figure 4a). The pseudovirions used were produced using the HIV-2-CRU5SIN-WPRE transducing vector instead of the HIV-2-CRU5SIN-CGW, thus, the fluorescence obtained was solely attributed to Tat-induced LTR transactivation, since the HIV-2-CRU5SIN-WPRE vector did not contain a CMV-driven GFP expression (Figure S3). Additionally, dot-blotting was used to confirm the presence of HIV-2 Tat in the pseudovirions (Figure 4b), and intracellularly from GHOST(3) cell lysate following transduction. β-actin was used as control (Figure 4c).

#### 2.3.2. Effects of Y44A and Y55A Mutations on RT Activity

**Figure 5:**
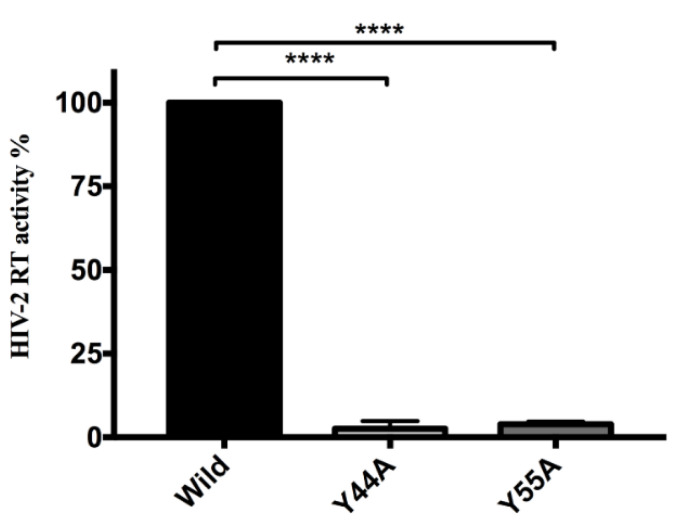
Tat mutations effect on HIV-2 reverse transcriptase activity. Quantification of HIV-2 RT. Activity of HIV-2 RT was significantly decreased in the presence of Tat mutations. Amount of pseudovirions have been normalized to capsid ELISA. Error bars represent SD (n = 6), **** *p* value < 0.0001.

An ELISA-based colorimetric reverse transcriptase assay was used to determine whether Y44A mutation had an effect on RT activity. Pseudovirions produced using vectors coding for the Y44A and Y55A Tat mutations had significantly diminished RT activity (3% and 4%, respectively) compared to that of the wild-type (*p* values < 0.0001), implying a detrimental effect of the mutations on the activity of RT (Figure 5).

**Figure 6:**
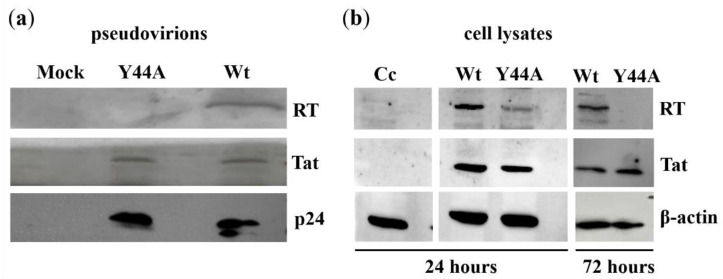
Tat mutations effect on HIV-2 reverse transcriptase expression. (**a**) Western blot analysis of RT, Tat and p24 in virions produced from transfected HEK293T cells. Viruses were concentrated by ultracentrifugation, pelleted viruses were lysed and viral proteins were resolved by SDS-PAGE and transferred onto nitrocellulose membranes. For protein detection, monoclonal anti-p24 (capsid) or polyclonal antibodies against HIV-2 Tat or HIV-2 RT were applied. (**b**) HEK293T cells were transfected for pseudovirion production, then lysed after 24 (D1) and 72 h (D3). Anti-Tat serum and polyclonal HIV-2 RT antibody were used to detect HIV-2 Tat and RT from transfected cell lysate, respectively. β-actin was used as control for cell lysate of transfected HEK293T cells. Western blot was repeated three times with similar results and the results of representative experiments are shown. Cc, non-transfected HEK293T cells.

A Western blot was then carried out in order to qualitatively determine whether the Y44A Tat mutation had any effect on the quantity of RT packaged into the virions. Interestingly, we were not able to detect RT in the presence of Y44A mutant Tat from the lysate of pseudovirions (Figure 6a). This finding explains why RT activity was abolished as a result of the mutation. The amount of capsid and Tat in the pseudovirions was not affected.

Consequently, we explored changes in RT as a result of the mutation using transfection experiments. We transfected HEK293T cells with wild-type and Y44A mutant Tat coding HIV-2 CGP plasmids, thereafter, we followed changes in RT quantity by Western blot of transfected cell lysate over a period of 3 days. After 24 h, we noticed that the amount of RT was lower in the presence of Y44A mutation compared to that found in the wild-type, and after 3 days, RT was minimally detected (Figure 6b). As a control, we have used β-actin.

#### 2.3.3. Detection of Tat in Pseudovirions

**Figure 7:**
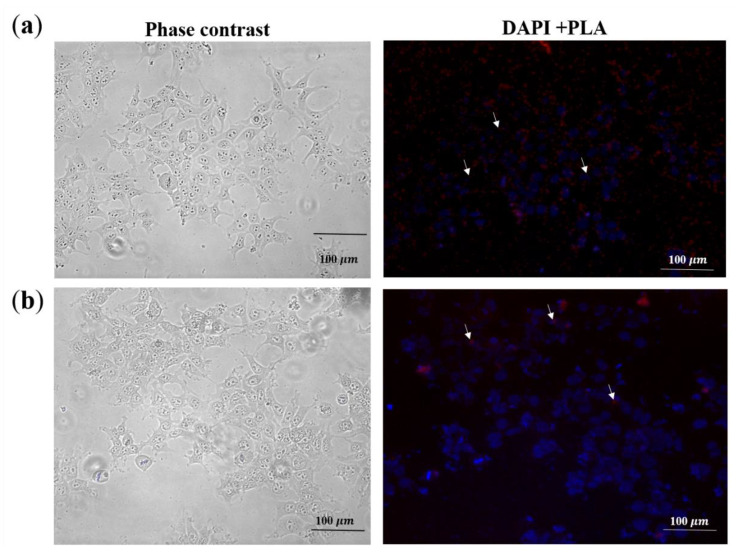
Detection of HIV-2 Tat was performed using PLA. HEK293T cells were transduced with 19 ng (normalized for capsid) (**a**) wild-type or (**b**) Tat Y44A mutant HIV-2. After 1 h incubation, cells were fixed, permeabilized and processed for PLA (proximity ligation assay). Cell nuclei were stained with DAPI. Scale bars: 100 µm. Each red spot represents a single interaction between HIV-2 Tat and Vpx. For representation, some of the PLA signals are indicated by white arrows. Phrase contrast of both wild-type and mutant HIV-2 Tat is represented as well.

To date, incorporation of Tat into the pseudovirions remains debated, and to our best knowledge it has not yet been definitively demonstrated that Tat becomes incorporated into HIV-1 virions, and exosomal expression is believed to greatly contribute to the expression of Tat [36]. Proximity ligation assay (PLA) was used to detect HIV-2 Tat in the pseudovirions. Using anti-Vpx and anti-Tat antibodies, we detected the fluorescence signal indicating interaction between the two viral proteins. Since Vpx is known to be incorporated into virions [37], this implied that Tat was similarly packaged into the pseudovirions (Figure 7a). Tat incorporation was also detected for mutant Tat carrying virions (Figure 7b). Additionally, we tested whether Tat can be detected in the supernatant, and for this, we conducted a mock transfection of the cells with a wild-type coding HIV-2 CGP plasmid. Western blot of the transfected cells supernatant in this case failed to detect the Tat protein (Figure 6a).

## 3. Discussion

While the effects of mutations on the function of HIV-1 Tat are well characterized, substitutions in HIV-2 Tat, especially in the acidic domain, are understudied.

The N-terminal region of the two proteins shows minimal homology, and mutations in the acidic domain of HIV-1 Tat are well tolerated and do not affect the protein’s function [26,38]. On the other hand, mutations in the core and/or basic region of HIV-1 Tat adversely affect reverse transcription [39], while effects of mutations in the Pro-rich domain of HIV-2 Tat are yet to be been defined. Here, we have characterized the Y44A mutation in HIV-2 Tat protein, and evaluated its effect on RT activity, and facilitation of proviral transcription in an indicator cell line.

Sequence-based predictions and stability analyses of homology model structures, available in public SWISS-MODEL repository, were used to map those residues in the central region of HIV-2 Tat which can be potentially targeted by a point mutation for protein inactivation. The target mutation was expected to influence the secondary structure of the putative ordered region and hence, impair the protein’s function. The applied models were considered to be potentially useful for structure-based mutation design, since the model structures have been prepared based on actual template selection (SWISS-MODEL Template Library version 9 August 2017). However, they showed 33.3% target-template sequence identity that is slightly above the sub-optimal template selection (<30%). Furthermore, the coverage of the target sequence was 73.9% (96/130 residues), and the model contained the conserved domains of interest. Based on stability analyses, Y44A was assumed to inactivate HIV-2 Tat, as we predicted a destabilizing nature of the mutation by multiple methods.

We then carried out a series of in vitro experiments to study the inactivating nature of the Y44A mutation and characterize its effect on reverse transcription and viral replication. For comparison, Y55A Tat mutant was also studied.

We carried out experiments on HIV indicator cell line to assess the effect of the Y44A and Y55A Tat mutations on transactivation of the proviral genome. A significantly decreased transactivation was observed when we used pseudovirions containing the Y44A and Y55A Tat mutations, compared to the wild-type protein. This was expected for the Y55A mutant, since Y26A mutation of HIV-1 Tat has been proved to reduce Tat-induced activation of HIV LTR [32], and Y55 residue of HIV-2 Tat corresponds the tyrosine in the 26th position of HIV-1 Tat. Nevertheless, our finding regarding the Y44A mutant confirms that mutation at the 44th residue also diminishes the activity of Tat. This result is in good agreement with that of previous studies, wherein the function of the Pro-rich domain was described, showing that the 38–48 region is critical for transactivation of the LTRs of HIV-2 and HIV-1. Moreover, deletion in the amino-terminal region (30–47 residues) was shown to result in a reduced LTR transactivation capability [25]. Additionally, deletion of 26–46 residues drastically reduced the activation function of HIV-2 Tat [30].

Interaction between Tat and RT has been described previously, as it was reported that HIV-1 Tat interacts with both p51 and p66 subunits of RT in vitro, thereby coordinating and stimulating reverse transcription [16]. Our results showed that Y44A mutation of Tat completely abolished RT activity, compared to the wild-type, akin to the Y55A mutation. Furthermore, Western blot of the pseudovirion lysate revealed that RT was not detectable in the presence of Y44A mutation. We hypothesized that the mutation might have interfered with the stability and packaging of the RT into the pseudovirions as others have described previously [40], therefore, we carried out transfection experiments using plasmids coding for the wild-type and Y44A Tat mutant, and utilized Western blot analysis of cell lysate to detect changes in RT during a 3 day period. While expression of the RT was clearly detectable in the case of the wild-type in all studied time points, in relatively equal amounts, we noticed that in the presence of Y44A Tat mutant, the amount of RT was significantly lower in the first day after transfection, and was almost undetectable on day 3 post-transfection (Figure 6b).

The possible mechanism behind the proposed stabilization of RT by Tat remains to be elucidated, and perhaps our results indicate degradation of the RT as a result of the Y44A Tat mutation. A previous study reported that the mutant of the two-exon HIV-1 Tat protein (Nullbasic) can directly bind RT affecting the RT complex and viral core stability [40], however, we have not explored this mechanism further, as it was beyond the scope of our study. The transcriptional activity of Tat is stimulated by ubiquitination, and Tat itself has been found to induce the polyubiquitination and proteasomal degradation of many cellular proteins in order to enhance viral pathogenesis [11]. It is therefore plausible that a mutation in the Pro-rich domain of HIV-2 Tat may in fact target RT for proteasomal degradation, in a ubiquitination-dependent or -independent manner.

Interestingly, we were able to prove that Tat is incorporated into the pseudovirions as indicated by our PLA assays. To our knowledge, the incorporation of Tat into virions has never been described previously, despite its ability to permeate cell plasma membranes [41]. Utilizing anti-Vpx and anti-Tat antibodies, a PLA fluorescence signal was obtained indicating the presence of Tat within the pseudovirions. To rule out the possibility that exosomal expression of Tat in the cell supernatant might have interfered with our assays, we collected the supernatant of cells transfected only with the HIV-2 CGP plasmid expressing Tat, and after following the same protocol used to produce the pseudovirions, Western blot failed to detect the viral protein. Isolation and analysis of exosome fraction may unequivocally reveal that the Tat protein is packed into pseudovirions but not into exosomes.

Given the concurrency of our in silico and in vitro results, our findings imply that the applied methods can be used for mutation analysis and design, but the possible uncertainties of the models, caused by relatively lower target-template identity, need to be considered, and sophisticated experimental approaches may be applied in future studies to confirm the predicted changes. The Y44A mutation was predicted to alter the structure of an ordered region, thereby inactivating the protein. This indicated that the Pro-rich domain has a main role in the transactivation function of HIV-2 Tat, unlike Tat protein of its counterpart. Furthermore, the findings of this study suggest that the first domain of HIV-2 Tat is involved in the regulation and stability of RT and reverse transcription, in addition to Tat-dependent LTR transactivation.

## 4. Materials and Methods

### 4.1. Data Acquisition and In Silico Predictions

Sequence of HIV-2 Tat protein was downloaded from UniProt database (UniProt ID: P04605), while model structures were acquired from the public SWISS-MODEL repository [42] (SWISS-MODEL Template Library identifiers: 1tvs.1 and 1tvt.1) (https://swissmodel.expasy.org/repository/uniprot//P04605, date of download: 27 March 2017). The downloaded models were prepared based on the coordinate files of equine infectious anemia virus Tat protein (PDB IDs: 1tvs and 1tvt). Full-length HIV-2 Tat sequences were downloaded from the Los Alamos National Laboratory (LANL) HIV sequence database [38] (https://www.hiv.lanl.gov/content/sequence/NEWALIGN/align.html, date of download: 28 October 2019) (Table S1). A multiple-sequence alignment of amino acid sequences (without any gap) was made using ClustalW (https://www.genome.jp/tools-bin/clustalw). The divergence of sequences was schematically visualized using Weblogo (for 1–70 residues, in the first two domains) (https://weblogo.berkeley.edu/logo.cgi).

IUPred2A webserver (https://iupred2a.elte.hu) was used for disorder prediction (prediction type: short disorder and structured regions) [43], I-Mutant 2.0 server for calculation of changes in stability upon point mutations [44], and JPred4 server for secondary structure prediction [45]. Site Directed Mutator (SDM) server [46] and FoldX [47] structure-based algorithms were applied to predict the effects of point mutations on protein stability.

### 4.2. HIV-2 Vector System

We utilized 2nd generation lentiviral vectors for HIV-2 pseudovirus production: (i) HIV-2 CGP vector as a structural HIV-2 protein expression construct which encodes all HIV-2 genes except *nef* and *env*; (ii) HIV-2-CRU5SIN-CGW vector as a minimal HIV-2 plasmid containing a green fluorescent protein (GFP) expression cassette under a CMV promoter; (iii) HIV-2-CRU5SIN-WPRE as a transducing vector that contains U5 regions and HIV-2 *gag* without CMV promoter or GFP, where this vector was used in experiments with HIV indicator cell line; (iv) pMD.G vector, which encodes for the envelope protein of vesicular stomatitis virus (VSV) [48].

HIV-2 CGP, HIV-2-CRU5SIN-CGW and HIV-2-CRU5SIN-WPRE were a kind gift from Joseph P. Dougherty at the Robert Wood Johnson Medical School (New Brunswick, NJ, USA) [49].

### 4.3. Mutagenesis

To generate the Y44A and Y55A mutations in HIV-2 Tat protein, HIV-2 *tat* gene, encoded by the HIV-2 CGP vector, was modified by site-directed mutagenesis using QuikChange Lightning Multi Site-Directed Mutagenesis Kit and QuikChange II XL Site-Directed Mutagenesis Kit (Agilent Technologies, Santa Clara, CA, USA), respectively. The following mutagenesis primers were used: Y44A_forward primer: 5′-CTC TCT CAG CTA GCC CGA CCC CTA GAA AC-3′, Y44A_reverse primer: 5′-GT TTC TAG GGG TCG GGC TAG CTG AGA GAG-3′Y55A_forward primer: 5′-CA TGC AAT AAC TCA TGC GCC TGT AAG CGA TGC TGC TAC CAT TG-3′, and Y55A_reverse primer: 5′-CA ATG GTA GCA GCA TCG CTT ACA GGC GCA TGA GTT ATT GCA TG-3′. PCR sequencing was performed to verify the success of mutagenesis.

### 4.4. Experiments on HEK293T Cells

#### 4.4.1. Production of Pseudovirions

To produce HIV-2 pseudovirions, HIV-2 CGP, HIV-2-CRU5SIN-CGW, and pMD.G plasmids were used at a ratio of 1:1:1 [34]. HEK293T cells (Invitrogen) were seeded in T-75 flask in 15 mL Dulbecco’s Modified Eagle’s Medium (DMEM) (Sigma-Aldrich, St. Louis, MO, USA) supplemented with 10% fetal bovine serum (FBS), 1% glutamine, and 1% penicillin-streptomycin.

24 h before transfection, HEK293T cells were passaged in order to achieve 70% confluency (5–6 × 10^6^ cells/mL) on the next day. A total of 30 µg plasmid DNA was used for transfection, using polyethylenimine (PEI) (Sigma-Aldrich, St. Louis, MO, USA). Cells were then incubated at 37 °C, 5% CO_2_ for 5–6 h in 5 mL DMEM supplemented with 1% FBS and containing no antibiotics. The medium was then replaced by 15 mL DMEM containing 10% FBS, 1% glutamine, and 1% penicillin-streptomycin. The supernatant was collected and filtered through a 0.45 µm polyvinylidene fluoride filter (Merck Millipore, Darmstadt, Germany) at 24, 48, and 72 h after transfection, and then concentrated by ultracentrifugation (100,000× *g*, 2 h, 4 °C). The pellet containing viral particles was then dissolved in 200 µL phosphate-buffered saline (PBS) and stored at −70 °C. ELISA-based colorimetric reverse transcriptase (RT) assay (Roche Applied Science, Mannheim, Germany) was then used to detect the amount of RT in the viral samples according to the manufacturer’s protocol. Concentration of HIV-2 capsid was measured using an ELISA-based colorimetric SIV p27 assay (Express Biotech International, Frederick, MD, USA).

#### 4.4.2. Transduction of HEK293T Cells

The day before transduction, HEK293T cells were plated in a 48-well plate in 300 µL of DMEM supplemented with 10% FBS, 1% glutamine, and 1% penicillin-streptomycin. At ~50% confluency (5.4 × 10^5^ cells/mL), cells were infected with 6 ng HIV-2 (normalized for p27) in 500 µL serum- and antibiotic-free media, supplemented with 8 µg/mL polybrene. On the next day, wells were complemented with 250 µL of DMEM containing 20% FBS, 2% glutamine, and 2% penicillin-streptomycin. The cells were thereafter incubated at 37 °C, 5% CO_2_ for 4 days. The media were then discarded and cells were mechanically suspended in 200 µL PBS. After brief centrifugation (5 min, 152× *g*), PBS was discarded and the cells were suspended in 500 µL PBS supplemented with 1% formaldehyde. For quantitative analysis, cells were counted by flow cytometry (FACSCalibur, BD Biosciences, Singapore) to determine the percentage of GFP-positive cells.

#### 4.4.3. Detection of HIV-2 Tat and RT by Western Blot

HEK293T cells were transfected with HIV-2 CGP plasmid coding for the wild-type or mutant Tat, HIV-2-CRU5SIN-CGW, and pMD.G plasmids as described in the “Production of pseudovirions” section. As control, HEK293T cells were transfected with 10 µg of HIV-2 CGP carrying wild-type HIV-2 *tat* in T75 flasks using PEI. After transfection the supernatant was collected and filtered through a 0.45 µm polyvinylidene fluoride filter (Merck Millipore, Darmstadt, Germany) after 24, 48, and 72 h of transfection, and then concentrated by ultracentrifugation (100,000× *g*, 2 h, 4 °C). Then the pellet containing viral particles was dissolved in 200 µL phosphate-buffered saline (PBS) and stored at −70 °C. 1 ng of the pseudovirions (normalized for p27 capsid) were suspended in lysis buffer (50 mM Tris, 80 mM KCl, 25 mM dithiothreitol (DTT), 0.75 mM ethylenediaminetetraacetic acid (EDTA), 0.5% Triton X-100, pH 7.8), thereafter incubated for 30 min at room temperature. Then, 40 µL of the lysate was then loaded on 12% SDS polyacrylamide gel. Primary antibodies, anti-p24 monoclonal antibody [50], Tat antiserum [51] and anti-HIV-2 RT [52] antibodies were used, followed by the use of secondary anti-mouse IgG for anti-p24 monoclonal antibody (Sigma-Aldrich, St. Louis, MO, USA), or anti-rabbit IgG (BioRad, Hercules, CA, USA) for anti-Tat and anti-RT antibodies. SuperSignal West Pico Chemiluminescent substrate (Thermo Fisher Scientific, MA, USA) was used to detect the bands.

In order to detect intracellularly expressed Tat and RT, cells were transfected with 10 µg of HIV-2 CGP plasmid coding for either the wild-type or Y44A mutant Tat. A total of 24 and 72 h after transfection, cells were mechanically scrapped in 5 mL ice cold PBS. Cells were then centrifuged for 10 min at 152× *g*, and the pellet was suspended in 1 mL ice cold PBS. After a brief sonication (Branson Sonicator, 3 × 2 min, 4 °C), the lysate was centrifuged for 30 min at 13,500× *g*, 4 °C). Then, 30 µL of the supernatant (normalized to β-actin) was loaded onto 10% SDS polyacrylamide gel. After blotting of proteins to nitrocellulose membrane, the membrane was incubated with either HIV-2 Tat antiserum or anti-HIV-2 RT antibody, followed by washing steps, incubation with secondary anti-bodies, and detection using the SuperSignal West Pico Chemiluminescent substrate.

#### 4.4.4. Proximity Ligation Assay

HEK293T cells were plated on polylisin pre-coated 8-well ibidi chambers (30,000 cells/well) (ibidi GmbH, Gräfelfing, Germany) in 300 µL DMEM supplemented with 10% FBS, 1% glutamine, and 1% penicillin-streptomycin. At ~50% confluency, cells were infected with 2 ng (normalized for p27) of wild-type and Y44A Tat mutant HIV-2 pseudovirions, in 200 µL serum- and antibiotic-free media, supplemented with 8 µg/mL polybrene. Cells were incubated at 37 °C, 5% CO_2_ for 1 h, then the supernatant was discarded and the cells were washed with 100 µL PBS, and fixed with 8% formaldehyde (Thermo Fisher) for 30 min at room temperature. Cells were then washed twice with 100 µL PBS, and permeabilized with PBS containing 0.5% Triton-X 100 for 10 min at room temperature. The PLA protocol was performed using Duolink PLA Kit (Sigma-Aldrich, St. Louis, MO, USA) and anti-Vpx [53] and anti-Tat antibodies at 1:100 dilutions according to the manufacturer’s recommendations. Interaction was detected by fluorescence microscopy using EVOS FLoid Cell Imaging Station.

### 4.5. Experiments on GHOST(3) Cells

#### 4.5.1. Production of Viral Particles for Transduction of GHOST(3) Cells

Production of pseudovirions for transduction of GHOST(3) parenteral cells (NIH AIDS Reagent Program; cat. no.: 3679) [54] was performed similarly to what was described in the “Production of pseudovirions” section, with the exception that we used the HIV-2-CRU5SIN-WPRE vector instead of HIV-2-CRU5SIN-CGW. HIV-2-CRU5SIN-WPRE is composed of U5 regions and HIV-2 *gag* without CMV promoter or GFP.

#### 4.5.2. Transduction of GHOST(3) Cells

The day before transduction, GHOST(3) parenteral cells were plated in a 12-well plate in 1000 µL DMEM supplemented with 7.5% FBS, 1% glutamine, and 1% penicillin-streptomycin (100,000 cells/well).

At approximately 50% confluency, GHOST(3) indicator cells were infected with 5 ng (normalized for p27) of HIV-2 pseudovirions containing either the wild-type or mutant Tat proteins in 1 mL of serum- and antibiotic-free media, supplemented with 2 µg/mL of polybrene. After 2 h incubation, the cells were supplemented with 500 µL of DMEM containing 7.5% FBS, 1% glutamine, and 1% penicillin-streptomycin, and were further incubated at 37 °C, 5% CO_2_ for 3 days. The medium was then discarded and cells were mechanically suspended in 200 µL PBS. After brief centrifugation (152× *g*, 5 min), PBS was discarded, and the cells were suspended in 500 µL of PBS supplemented with 1% formaldehyde. FACS analysis was then carried out to quantify the percentage of LTR-induced GFP positive cells. The protocol of transduction was adopted and modified from Vödrös et al. [55].

#### 4.5.3. Dot-Blotting

In order to detect HIV-2 Tat in the harvested pseudovirions, 1 ng of the virions (normalized for p27) was suspended in lysis buffer (50 mM Tris, 80 mM KCl, 25mM DTT, 0.75 mM EDTA, 0.5% Triton X-100, pH 7.8), and thereafter incubated for 30 min at room temperature. Then, 20 µL of virus lysate was loaded onto nitrocellulose membrane. Primary antibodies, β-actin antibody (Covalab, Villeurbanne, France) and HIV-2 Tat antiserum were used, followed by the use of secondary antibodies. Dot blots were detected using SuperSignal West Pico Chemiluminescent substrate.

Dot-blotting of the cell lysate was performed as follows: GHOST(3) cells were transduced with 5 ng (normalized for p27) of wild-type or Y44A/Y55A mutant Tat HIV-2 pseudovirions as described previously. After 3 days incubation, the medium was discarded, and cells were mechanically scrapped in 3 mL of ice-cold PBS. Cells were then centrifuged for 6 min at 152× *g*, and the pellet was suspended in 500 µL lysis buffer (50 mM Tris-HCl, 250 mM NaCl, 0.5% NP-40, 5 mM EDTA, 50 mM NaF, pH 7.4). Thereafter, the dissolved pellet was placed on ice for 30 min, and vortexed every 10 min. After sonication, 20 µL of the cell lysate was loaded onto nitrocellulose membrane (normalized to β-actin).

### 4.6. Statistical Analysis

Comparison of RT activities, transduction efficiencies, and LTR-induced GFP activities of wild-type and Tat-mutants were performed by one-way ANOVA analysis using GraphPad Prism 7.0 (GraphPad Software, San Diego, CA, USA).

## Supplementary Materials

Supplementary Materials can be found at https://www.mdpi.com/1422-0067/21/16/5907/s1.

## Abbreviations

AIDS	acquired immunodeficiency syndrome
CMV	cytomegalovirus
HIV	human immunodeficiency virus
HIV-2	human immunodeficiency virus type 2
GFP	green fluorescent protein
LTR	RNA element on the long terminal repeat
MHC	major histocompatibility complex
PLA	proximity ligation assay
P-TEFb	positive transcription elongation factor b
RNAPII	RNA polymerase II
RT	reverse transcriptase
SIV	simian immunodeficiency virus
TAR	transactivation response
Tat	transactivator protein
Vpx	Viral protein X
