# Supplementary material for: Y44A Mutation in the Acidic Domain of HIV-2 Tat Impairs Viral Reverse Transcription and LTR-Transactivation
Source: Int J Mol Sci. 2020 Aug 17;21(16):5907. doi: 10.3390/ijms21165907 (PMC7460587; doi:10.3390/ijms21165907)
Supplement: Supplementary file 1 [file ijms-21-05907-s001.zip › 866339-Table-S1.pdf]

Table S1. Polymorphism of HIV-2 Tat sequences in the first two domains of the protein. Accession number, date and country of isolation, protein ID, and sequence name is shown for all sequences.

**Accession number**	**Name**	**Subtype**	**Isolation year**	**Isolation country**	**Protein ID**
U38293	A.CI.88.UC2	A	1988	Cote d'Ivoire	AAB47786.1
M30502	A.DE.x.BEN	A	1990	Germany	AAB00741.1
U22047	A.DE.x.PEI2_KR_KRCG	A	1995	Germany	AAA64580.1
KY025539	A.FR.93.LA37	A	2000	France	APJ01780.1
Z48731	A.GW.x.MDS	A	2006	Guinea-bissau	CAA88625.1
MF595856	A.NL.01.RH2.13	A	2001	Netherland s	ATU79188.1
EU980602	A.IN.07.NNVA	A	2007	India	ACH73025.1
DQ307022	A.IN.95.CRIK_147	A	1995	India	ABC39622.1
AB731742	A.JP.08.NMC786_clone_41	A	2008	Japan	BAM76177.1
AF082339	A.PT.x.ALI	A	1998	Portugal	AAC95345.1
M15390	A.SN.85.ROD	A	1985	Senegal	AAB00768.1
L17625	B.CI.88.UC1	B	1988	Cote d'Ivoire	AAA43940.1
AB485670	B.CI.x.20_56	B	2009	Cote d'Ivoire	BAH97699.1
U27200	B.CI.x.EHO	B	1994	Cote d'Ivoire	AAC54471.1
KY025545	B.FR.00.LA44	B	2000	France	APJ01830.1
KY025544	B.FR.98.LA43	B	1998	France	APJ01822.1
AB100245	B.JP.01.IMCJ_KR020_1	B	2001	Japan	BAC79371.1
KP890355	F.US.08.NWK08	F	2008	United States	ALA65441.1
AF208027	G.CI.92.Abt96	G	1992	Cote d'Ivoire	AAF82033.1
EU028345	AB.CM.03.03CM_510_03	AB	2003	Cameroon	ABV83030.1
L36874	H2_01_AB.CI.90.7312A	H2_01_A B	1990	Cote d'Ivoire	AAL31357.1
AB731738	H2_01_AB.JP.04.NMC307_2 0	H2_01_A B	2004	Japan	BAM76141.1
AB731740	H2_01_AB.JP.07.NMC716_0 1	H2_01_A B	2007	Japan	BAM76159.1
AY530889	U.FR.96.12034	U	1996	France	AAT37067.1
