# Supplementary material for: Y44A Mutation in the Acidic Domain of HIV-2 Tat Impairs Viral Reverse Transcription and LTR-Transactivation
Source: Int J Mol Sci. 2020 Aug 17;21(16):5907. doi: 10.3390/ijms21165907 (PMC7460587; doi:10.3390/ijms21165907)

Figure S1. Genetic diversity of N-terminal Pro-rich acidic and Cys-rich domains of HIV-2 Tat. HIV-2 Tat sequences of group A, B (epidemic) and non-epidemic groups were collected from the Los Alamos National Laboratory (LANL) HIV sequence database (2019. 10. 28). A multiple sequence alignment of amino acid sequences was performed using ClustalW, while divergence of sequences was schematically visualized using Weblogo. Sequence logos show the Tat amino acid diversity for 1-70 residues of all HIV-2 groups (S3 A), epidemic groups A and B (S3 A and B) and non-epidemic groups of HIV-2 (S3 D). Red arrows indicate residues in 44th and 55th positions.

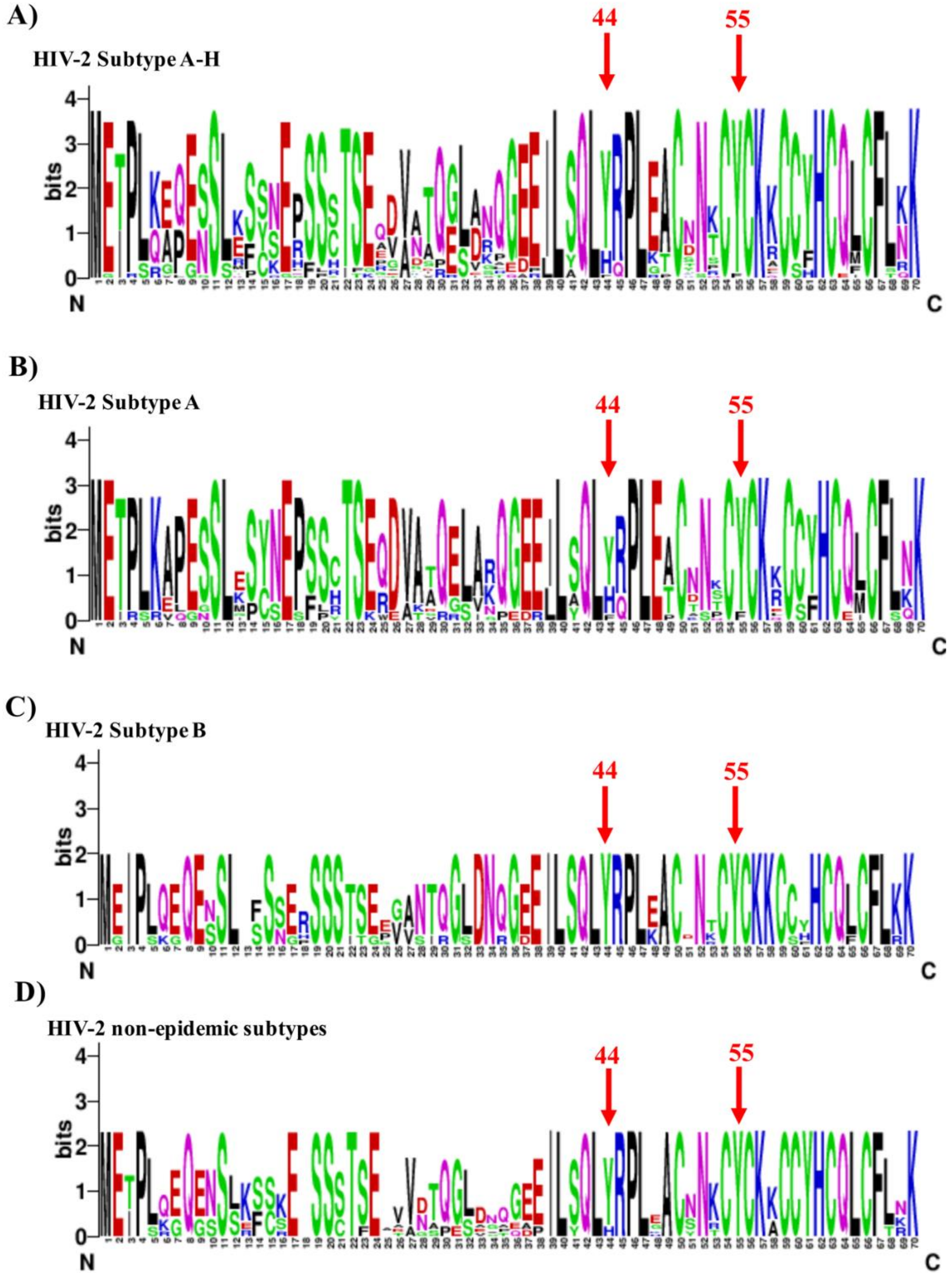

Supplement: Supplementary file 1 [file ijms-21-05907-s001.zip › 866339-Figure-S1.pdf]
