# Supplementary material for: Y44A Mutation in the Acidic Domain of HIV-2 Tat Impairs Viral Reverse Transcription and LTR-Transactivation
Source: Int J Mol Sci. 2020 Aug 17;21(16):5907. doi: 10.3390/ijms21165907 (PMC7460587; doi:10.3390/ijms21165907)

Figure S2. Transduction efficiency of Tat mutant HIV-2 pseudovirions on HEK293T cells. HEK293T cells were transduced by using the same amount of pseudovirions (6 ng, as normalized by capsid ELISA) (n=3). Percentage of fluorescent positive cells in the presence of wild-type Tat, and mutant Tat and protease were measured by flow cytometry. Active site mutant protease (PR) was used as a negative control. In the presence of Tat Y44A and Y55A substitutions, percentage of fluorescent cells did not change significantly compared to the wild-type HIV-2 Tat. Wt: wild-type, PR: active site mutant protease.

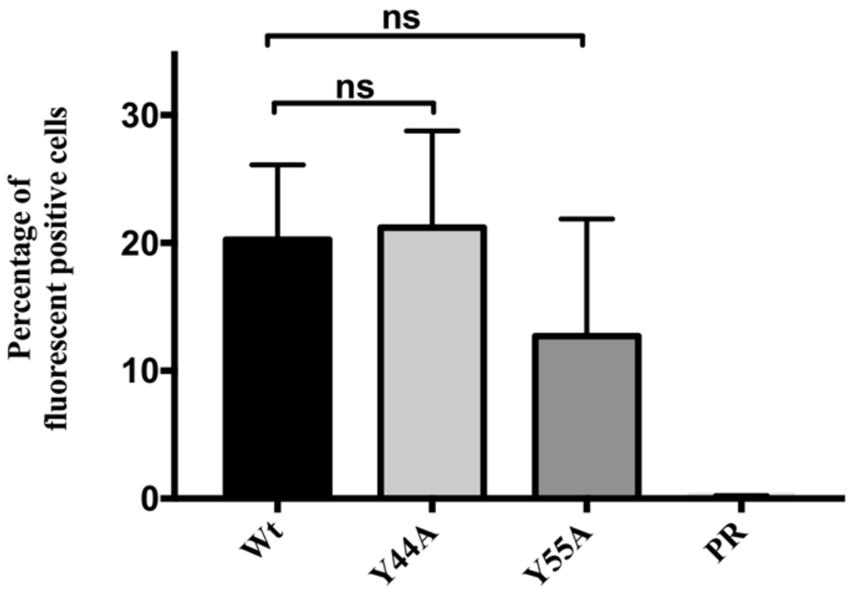

Supplement: Supplementary file 1 [file ijms-21-05907-s001.zip › 866339-Figure-S2.pdf]
