# Supplementary material for: Y44A Mutation in the Acidic Domain of HIV-2 Tat Impairs Viral Reverse Transcription and LTR-Transactivation
Source: Int J Mol Sci. 2020 Aug 17;21(16):5907. doi: 10.3390/ijms21165907 (PMC7460587; doi:10.3390/ijms21165907)

Figure S3. Transducing vectors used for HIV-2 pseudovirion production. **a**) CRU5SIN-CGW is an HIV-2-based minimal vector with a green fluorescent protein expression cassette under a CMV promoter. CRU5SIN-CGW vector was used in experiments with HEK293T cells. **b**) HIV-2-CRU5SIN-WPRE is a transducing vector that contains U5 regions and HIV-2 *gag*, without coding for green fluorescent protein. HIV-2-CRU5SIN-WPRE plasmid was used in experiments with GHOST(3) indicator cells. BGHpA: bovine growth hormone polyadenylation signal; CMV: human cytomegalovirus immediate early promoter; cppt: central polypurine tract; GFP: green fluorescence protein; RRE: rev response element; SD: splice donor site; R U5 and ΔU3 LTR: retroviral long terminal repeats; WPRE; woodchuck hepatitis virus post-transcriptional regulatory element.

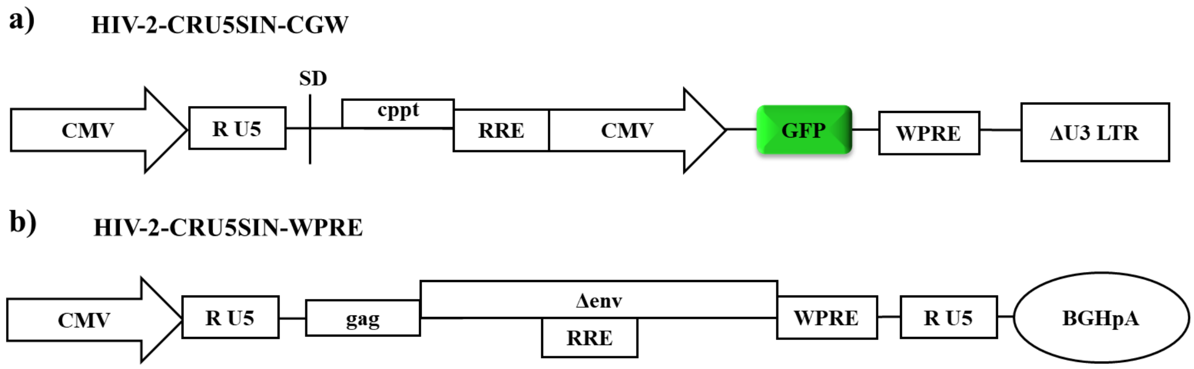

Supplement: Supplementary file 1 [file ijms-21-05907-s001.zip › 866339-Figure-S3.pdf]
